# Anterior segment optical coherence tomography findings of iris granulomas in Hansen's disease: a case report

**DOI:** 10.1186/1869-5760-3-36

**Published:** 2013-02-11

**Authors:** Padmamalini Mahendradas, Kavitha Avadhani, Sarika Ramachandran, Sahana Srinivas, Madhavi Naik, K Bhujang Shetty

**Affiliations:** 1 Department of Uveitis and Ocular Immunology, Narayana Nethralaya, 121/C, Rajajinagar, Bangalore, India; 2 Department of Dermatology, St Theresa's Hospital, Rajkumar Road, Rajajinagar, Bangalore, India; 3 Department of Pathology, Theresa's Hospital, Rajkumar Road, Rajajinagar, Bangalore, India

**Keywords:** Leprosy, Sclerouveitis, Anterior segment optical coherence tomography, ASOCT, Iris granuloma

## Abstract

**Background:**

A 50-year-old male was diagnosed to have a right eye sclerouveitis and left eye granulomatous anterior uveitis due to Hansen's disease. We are reporting the anterior segment optical coherence tomography (ASOCT) findings of iris granuloma in this case.

**Findings:**

Skin biopsy revealed plenty of acid fast bacilli with a bacteriological index of 5 suggestive of multibacillary polar lepromatous leprosy. ASOCT revealed well-demarcated smooth-surfaced nodular lesion with internal hyporeflectivity corresponding to the areas of granuloma which decreased in size following treatment with antileprosy drugs and systemic and topical steroids.

**Conclusion:**

ASOCT is a non-invasive technique to assess the extent of involvement of anterior segment in Hansen's disease and is a useful tool in follow-up. This is also the first report on ASOCT findings of iris granuloma in Hansen's disease.

## Findings

### Introduction

Leprosy (Hansen's disease or Hanseniasis) is one of the systemic diseases which can give rise to a plethora of features in the eye. The incidence of ocular involvement in leprosy has been variously quoted as 51% to 69% [1-3]. Iris nodules and lepra pearls are characteristics of the ocular manifestation of this disease and help in its diagnosis. In this study, we report the anterior segment optical coherence tomography (ASOCT) findings of iris granulomas in leprosy.

**Figure 1:**
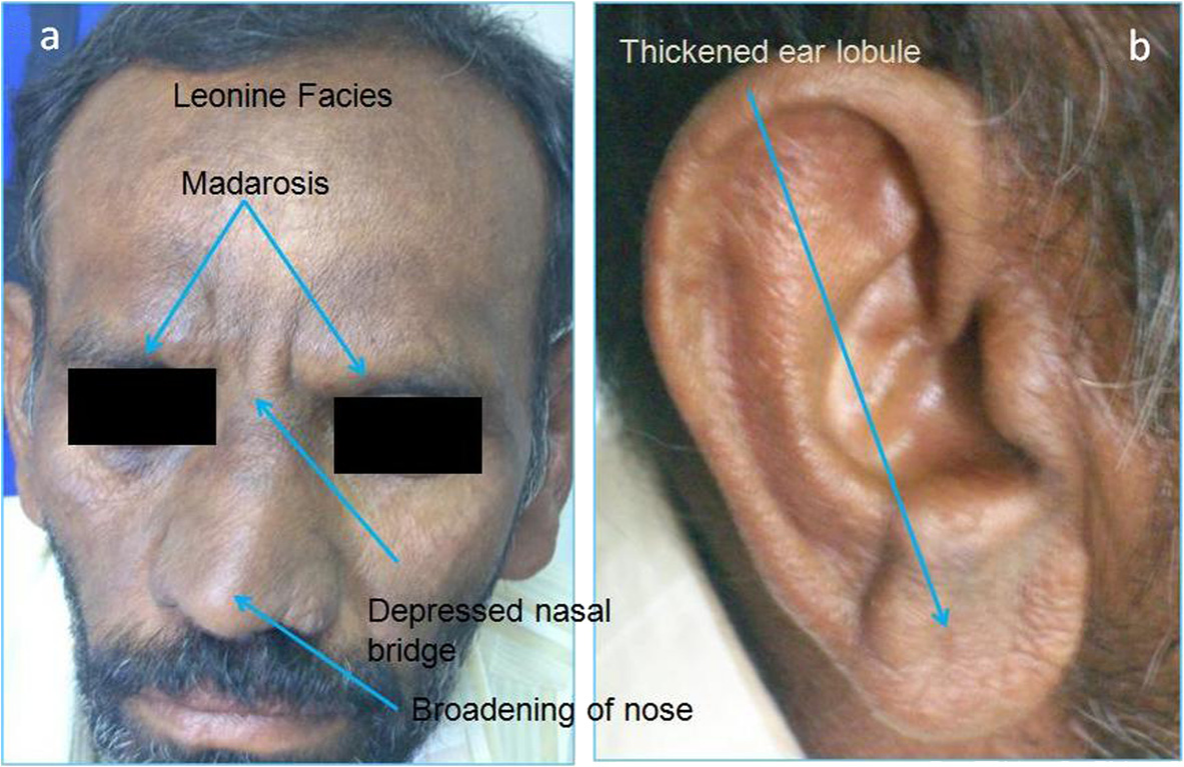
The face photograph of a 50-year-old man with leonine facies (a) and thickened ear lobule (b).

A 50-year-old gentleman presented to us complaining of watering in the right eye (OD) for 1 year, redness and diminution of vision for both distant and near for 2 months, bilateral headache and eye pain of 15-day duration. He was a known diabetic on medications for the past 1 year. He also gave history of undergoing treatment for dry, scaly skin lesions over the extremities in the past. On general physical examination, he had characteristic leonine facies with madarosis, depressed nasal bridge and broadening of the nose (Figure 1a). He also had thickened ear lobules (Figure 1b) and dry scaly patches of the skin over his extremities. His back showed bilaterally symmetrical patches of raised hypopigmented skin lesions.

**Figure 2:**
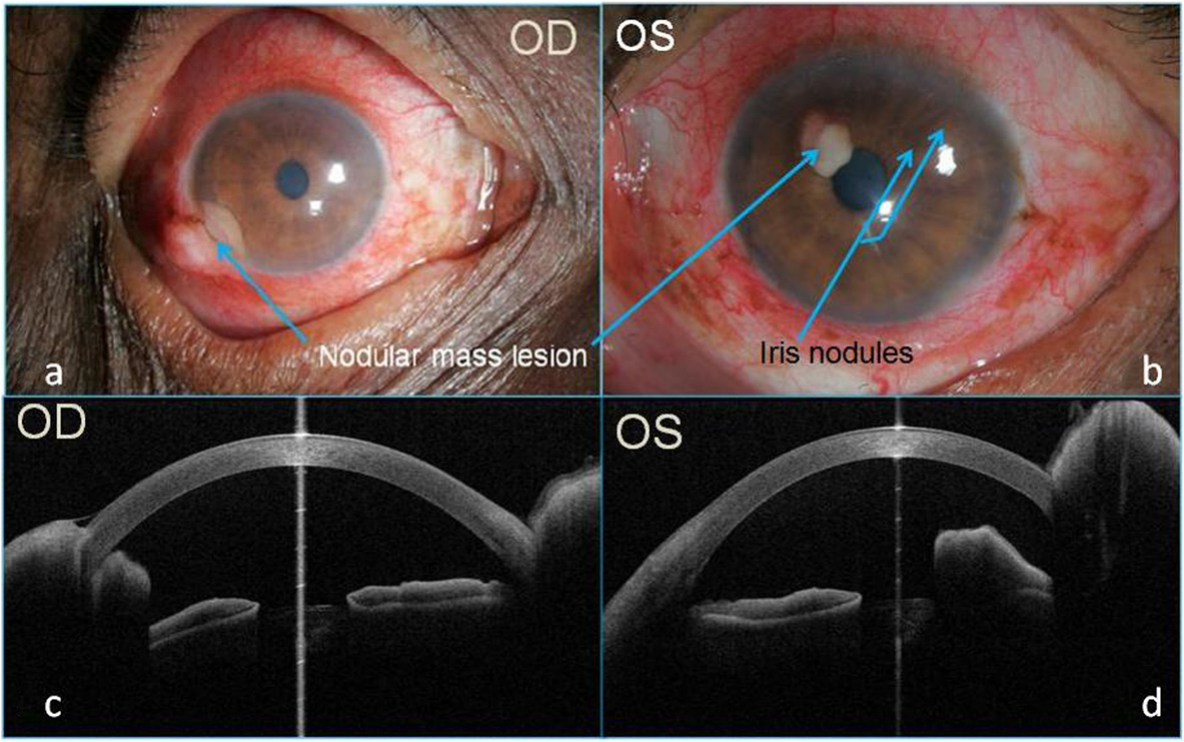
Granulomas in the eyes (a, b) and corresponding ASOCT findings (c, d).

On ocular examination, his best corrected visual acuity was 6/9, N10 in the right eye and 6/9, N8 in the left eye (OS). A nodular scleral mass lesion was noted in the right eye, adjacent to the limbus, around 3 × 2 mm in size, extending into the anterior chamber (Figure 2a). Other findings in the same eye were the presence of circumciliary congestion, fine keratic precipitates, 2+ grade flare and cells with prominent iris vessels. In the left eye, a nodular mass lesion arising from the iris (Figure 2b) adjacent to the pupillary border, 1 × 1 mm in size, was present. Iris pearls were also seen in the same eye along with circumciliary congestion, fine keratic precipitates, 2+ grade flare and cells and prominent iris vessels. Fundus examination of both eyes was found normal. A clinical diagnosis of the right eye granulomatous sclerouveitis and the left eye granulomatous anterior uveitis secondary to polar lepromatous leprosy was made (Figure 2). ASOCT (Cornea/Anterior Segment OCT SS-1000, Tomey Corporation, Nagoya, Japan) was carried out in order to document the extent of lesion. Imaging of the right eye showed a well-demarcated smooth-surfaced nodular lesion, with internal hyporeflectivity and after shadowing, involving the limbal sclera extending on to the cornea and into the angle and anterior chamber (Figure 2c). The left eye imaging showed a well-demarcated nodular lesion arising from the anterior surface of the iris with hyporeflective internal echoes corresponding to the areas of iris granulomas (Figure 2d). Two other well-circumscribed smaller nodular lesions were present on the iris and were observed on imaging.

**Figure 3:**
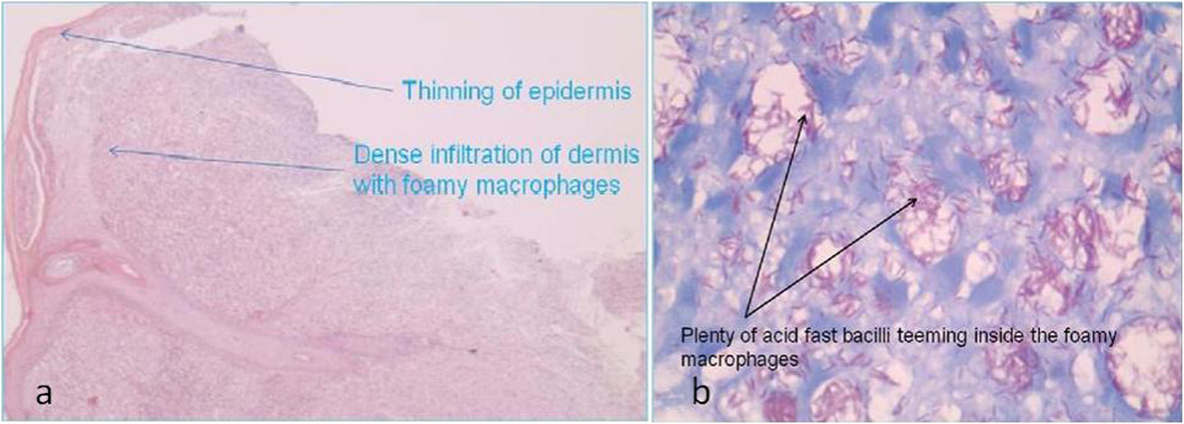
HPE shows foamy macrophages (a) and numerous acid fast bacilli (b).

Routine blood and urine investigations, mantoux, chest X-ray and HIV I and II tests were all found to be negative. Slit skin smears taken from both ear lobules were found to be positive for lepra bacilli. Biopsy specimen taken from the raised papular lesion on the back showed thinning of the epidermis and dense infiltration of the dermis with foamy macrophages (Figure 3a). Specimen from the same lesion stained with Fite-Faraco stain and viewed under oil immersion (×100 magnification) showed plenty of acid fast bacilli teeming inside the foamy macrophages (Figure 3b). The bacteriological index was found to be 5 (100 to 1,000 bacilli per high-power field). This bacteriological index put him in the category of multibacillary polar lepromatous leprosy.

**Figure 4:**
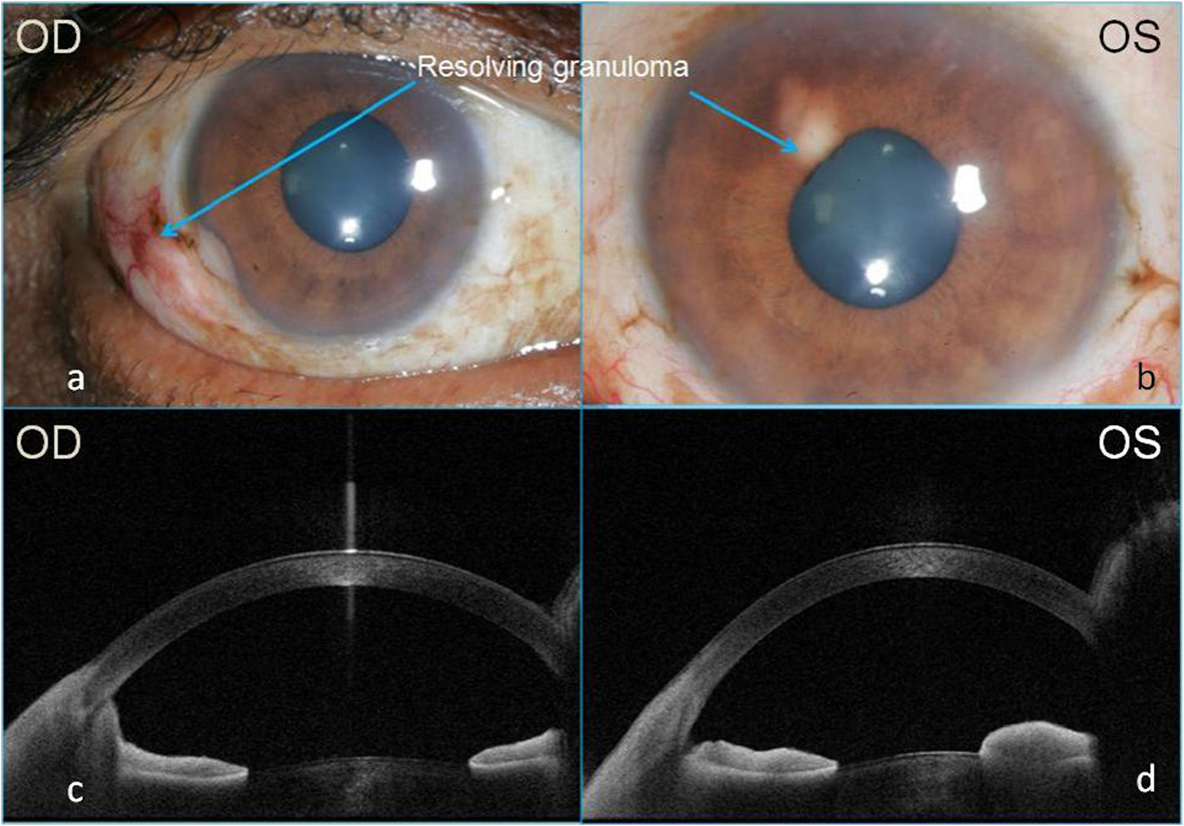
Resolving granulomas in the eyes (a, b) and corresponding ASOCT changes (c, d).

**Figure 5:**
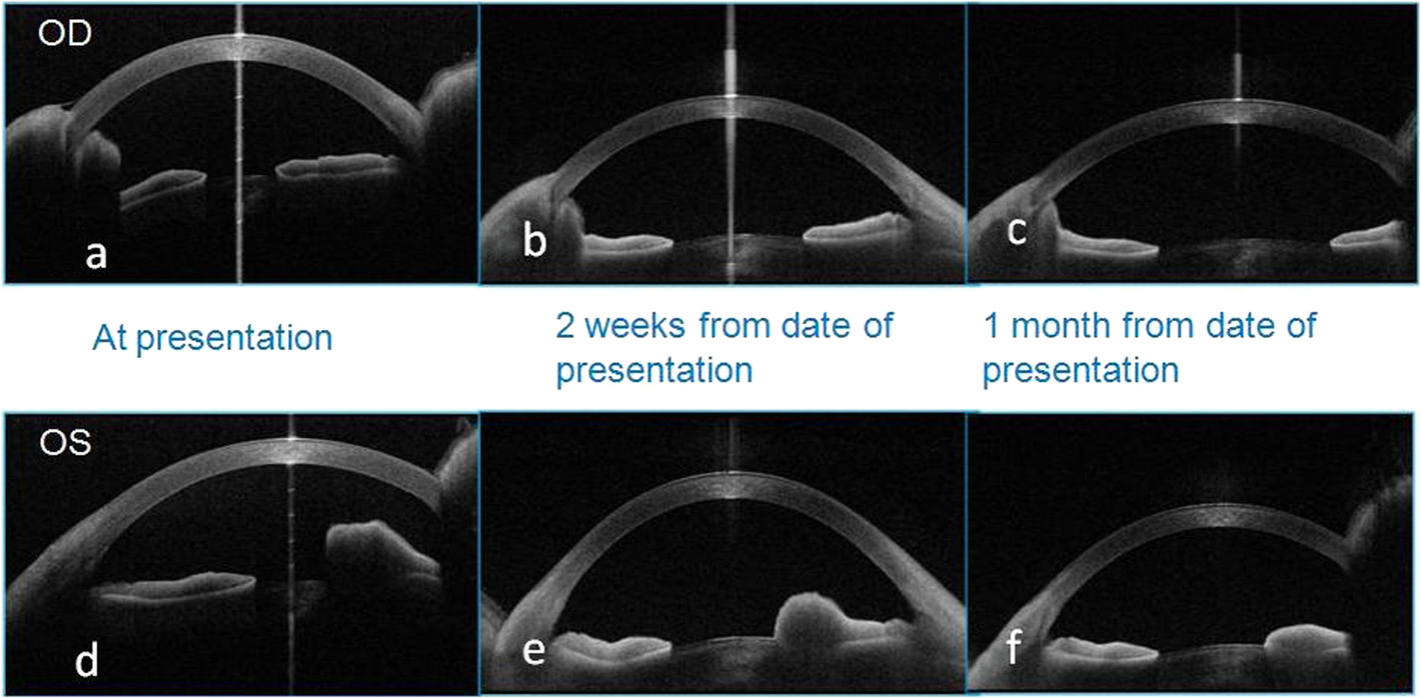
ASOCT features of iris granulomas at presentation (a, d), 2 weeks (b, e) and 4 weeks (c, f) post-treatment.

He was put on the standard anti-leprosy systemic regime comprising of dapsone, rifampicin and clofazamine with systemic steroids (Figure 4). In addition, he was started on topical steroids and cycloplegics for the ocular manifestations. Following treatment, his vision improved in his right eye, the nodular lesion and the iris pearls started resolving and the keratic precipitates had also reduced. One month after starting the treatment, the patient was asymptomatic and the eyes were quiet with the lesions reduced in size (Figure 4). Resolving lesions in both eyes were documented on ASOCT (Figure 5,b,c,e,f). At the end of 2 months, lesions in both eyes healed completely. Written informed consent was obtained from the patient for publication.

## Discussion

Ocular features commonly reported in leprosy are madarosis; lid abnormalities such as ectropion, entropion, trichiasis and lagophthalmos; dacryocystitis; chronic conjunctivitis; scleritis or episcleritis; and corneal involvement in the form of loss of corneal sensations, superficial keratitis, corneal opacities, interstitial keratitis, adherent leucoma, corneal ulcer or pannus and uveal involvement [2-5]. Three different types of uveal involvement have been described [5]: (a) typical acute granulomatous iridocyclitis with keratic precipitates, posterior synechiae and hypopyon with or without conjunctival leproma; (b) neuroparalytic uveitis in the form of dilator muscle atrophy; and (c) presence of iris pearls and lepra pearls with complicated cataract. Ocular hypotony in conjunction with hypotension has been reported in patients under therapy for multibacillary leprosy [6].

Ocular features noted in our patient were sclerouveal granuloma (leproma) with acute iridocyclitis in the right eye and iris leproma and iris pearls with acute iridocyclitis in the left eye. This case can be classified as type IV - immune-mediated granulomatous anterior uveitis due to leprosy [7]. Cornea was found to be the most commonly affected structure in the eye in a case series studied by Sanjiv et al. [8], of which loss of corneal sensations was the most common finding which in our case was notably absent. Lagophthalmos is another common ocular finding which was absent in this case. Ocular leproma can be secondary to taking dapsone monotherapy irregularly and can signify dapsone resistance [9]. Incidentally, our patient gave history of having taken some medications for a few months for the dry scaly skin lesions which he discontinued by himself.

ASOCT imaging of granulomatous lesions has been described earlier in tuberculosis as poorly demarcated amorphous lesion [10] and as absorbent, hyporeflective areas [11]. The lesions in our case were well demarcated with internal hyporeflectivity and after shadowing. The extent of involvement was easily made out on ASOCT, but the limitation of optics disallows the interpretation of depth of lesion.

## Conclusions

Granulomatous ocular leproma can be secondary to irregular anti-leprotic therapy or its discontinuation. Systemic steroids may be necessary along with anti-leprotic therapy for Immune-mediated granulomatous anterior uveitis seen in leprosy. ASOCT acts as a non-invasive technique to gauge the extent of involvement of anterior segment and is a useful tool for follow-up in patients of Hansen's disease with uveal involvement.

## Competing interests

The authors declare that they have no competing interests.

## Authors’ contributions

PM contributed to the design, acquisition and interpretation of data, and drafted the manuscript. KA contributed to the acquisition of data and drafted the manuscript. SR contributed to the acquisition and interpretation of data, and drafted the manuscript. SS contributed to the acquisition, interpretation and management of the skin biopsy. MN contributed to the interpretation of the skin biopsy and drafted the manuscript. BS contributed to the collection of data, critical revision of the manuscript and funding. All authors read and approved the final manuscript.
